# What are barriers and facilitators in sustaining lean management in healthcare? A qualitative literature review

**DOI:** 10.1186/s12913-023-09978-4

**Published:** 2023-09-06

**Authors:** Y. S. Kunnen, O. P. Roemeling, E. Smailhodzic

**Affiliations:** 1 Faculty of Economics and Business, University of Groningen, Groningen, the Netherlands; 2 Department of Innovation Management and Strategy, University of Groningen, Nettelbosje 2, Groningen, 9700 AV the Netherlands

**Keywords:** Healthcare, Continuous improvement, Lean management, Sustainability, Literature review, Qualitative methodology

## Abstract

**Background:**

Lean management (LM) is a continuous improvement methodology originating from manufacturing and is widely adopted in healthcare to improve processes. LM shows promising results in healthcare and research on the topic is increasing. However, it can be difficult to sustain LM over time, and an overview of facilitators or barriers that influence the sustainment of LM in a healthcare context is unavailable.

**Methods:**

Prior to search, five inclusion and exclusion criteria were defined to establish suitability of identified articles for our research question. This study was based on 24 selected peer-reviewed studies that reported on the sustainment of LM in healthcare organisations, published in the last five years. Following the Preferred Reporting Items for Systemtic Reviews and Meta-Analyses (PRISMA) guidelines, all articles were scanned, retrieved for full-text and analysed thematically.

**Results:**

Following thematic analysis, we identified four overarching themes: Mobilising Employees, Guiding Change Efforts, Methods, and Local Context. Key facilitators for supporting LM are fostering an improvement culture and learning culture, providing professional development opportunities, assigning more responsibilities to employees in decision making processes and appointing change agents to act as local LM leaders. Key barriers for sustaining LM include overburdening employees with responsibilities, omitting staff involvement during LM implementation, lack of patient engagement, lack of resources to engage with LM, a lack of leadership commitment and follow-up on projects, and a lack of knowledge of LM among leaders.

**Conclusion:**

Overall, studies emphasise the importance of actively involving and engaging the workforce to embed LM into organisational culture. Reflecting on the origins of LM, healthcare organisations can find inspiration in the virtue of respecting people in their journey to sustain and cultivate an improvement culture. LM provides potential to change healthcare for the better and could help healthcare organisations to cope with increasing external pressures.

**Supplementary Information:**

The online version contains supplementary material available at 10.1186/s12913-023-09978-4.

## Background

Healthcare organisations (HCOs) worldwide are under growing external pressure to become more efficient in containing or reducing healthcare costs while delivering the same or better quality care. In this study, we refer to HCOs as any kind of institution, both private and public, that is responsible for the provision of healthcare. Efficiency in HCOs may increase via adopting existing quality improvement concepts and methodologies commonly used in the manufacturing or services industry [1–3]. One of the methods often applied is lean management (LM). This continuous improvement methodology is increasingly being adopted by HCOs [4, 3, 5].

Through LM, one strives for perfection by continuously improving existing processes and integrating such an approach into organisational culture [1]. Consequently, this culture can be characterised as one in which all individuals or teams within an organisation work together to continuously improve processes and reduce errors, hence improving overall performance [6]. Thus, one can argue that continuous improvement results from sustained LM adoption in HCOs [1, 7]. The sustainable adoption of LM can be considered LM maturity [8], which can be assessed using the three-stage CI model developed by Fryer, Ogden and Anthony [6]. They state that CI, such as LM, can be considered adopted when embedded in organisational culture and are integral to administrative operations.

Prior studies that focused on LM sustainability in non-healthcare environments have shown the importance of developing a suitable organisational culture [9]. In addition, similar to our study, we witness research focusing on sustainability in terms of LM maturity, and highlighting the importance to focus on both process improvement and capability development [10]. Moreover, Santos and Tontini [11] developed a measure for LM maturity, focused on production environments, with elements such as supplier integration and measures focused on new product launches and stock turnover.

However, the specific attention to production settings in these prior studies does not easily translate to healthcare environments. The unique nature of LM in healthcare, where patients themselves are transformed in the healthcare process, requires its own research in order to understand sustainability of LM in the healthcare domain. We follow the reasoning of Radnor et al. [3] who argue healthcare has several traits which complicate the transfer and application of management principles, even when these have been shown to be effective in other sectors.

Reviews on LM in healthcare associate the implementation of LM with increased organisational effectiveness and cost-efficiency [12–14], though a few studies report contrasting findings [1, 15]. However, few studies have addressed the sustainability of LM post-implementation in HCOs [12], which is crucial for continuing CI programmes [16]. Little concrete evidence establishes whether LM efforts are sustained over time [12], and what barriers and facilitators exist to sustain LM over time remains underinvestigated [3, 13, 14].

A recent review by Flynn et al. [7] found that staff engagement, staff empowerment, and sense-making of LM may facilitate or hinder the sustainment of LM in paediatric healthcare. Another factor that may be important to sustaining LM is realising that successful implementation of LM is a long-term programme, not a short-term process improvement tool [3]. Naik et al. [17] identified that clear communication, the appointment of change agents, and facilitating training on LM might help sustain LM. In short, extant research identified or suggested barriers and facilitators for the sustainment of LM. However, an overview of facilitators and barriers that influence the sustainment of LM in a healthcare context remains unavailable.

To the best of our knowledge, this article is the first systematic literature review that focuses explicitly on identifying barriers and facilitators that influence sustaining LM in HCOs. Whilst prior studies have touched upon the topic in other domains, the attention to the healthcare context warrants its own research. Healthcare is typified by strong regulations from governmental bodies and is high in information asymmetry between providers and patients [18, 19]. Moreover, supply chains in healthcare are characterised by uniqueness and complexity [20]. Consequently, lessons from other environments such as manufacturing, do not translate well to our specific context.

This study has two objectives. First, we provide an overview of existing LM literature by systematically aggregating studies that report on sustaining LM. Second, we also develop a conceptual framework to visualise the relationships between barriers and facilitators that influence sustaining LM in HCOs. Accordingly, this study aims to answer the following research question: ‘*What are known barriers and facilitators to a sustainable implementation of ‘Lean management’ in healthcare?’.*

As discussed earlier, sustaining LM is crucial for continuing CI programmes and it remains a key challenge [16]. Identifying barriers and facilitators to sustain LM may aid HCOs in coping with the external pressures to contain or reduce healthcare costs and improve overall performance. Furthermore, addressing respective barriers or facilitators may allow for the sustainment of LM to occur over time [21]. This study contributes to the existing literature by developing a conceptual framework of barriers and facilitators to sustaining LM in healthcare. In addition, it also bears practical implications as it provides practitioners with a tool to guide and sustain quality improvement initiatives in practice.

## Methods

A systematic review focuses on identifying, evaluating, and synthesising literature [22] and reports findings in a systematic, explicit, reproducible and comprehensive manner [23]. In line with established practice, we followed the Preferred Reporting Items for Systematic Reviews and Meta-Analysis (PRISMA) guidelines [24] to conduct this systematic literature review.

### Search strategy

#### Databases

This review draws on twelve healthcare management journals that have been identified as valuable outlets for research in the healthcare management domain [25], see Table 1. With the selection of these sources, we aimed to increase the applicability and generalisability of findings to a broader audience. Moreover, we have focused on a subset of journals to cover a variety of perspectives, such as policy (e.g. Health Affairs), management and business (e.g. Healthcare Management Review), medical and quality (e.g. BMJ Quality and Safety), and social (e.g. Social Science and Medicine).

**Table 1:** Healthcare management journals that publish high quality research as reported by Meese et al. [25]

Health Care Management Review
Health Affairs
Social Science and Medicine
Health Services Research
Health Policy
Journal of Healthcare Management
Academy of Management Journal
Journal of Health Organisation and Management
BMJ Quality and Safety
Health Services Management Research
New England Journal of Medicine
Journal of the American Medical Association

#### Inclusion and exclusion criteria

Prior to commencing the literature search, five inclusion and exclusion criteria were defined. First, given the recent increase in publications on LM in healthcare (see D’Andreamatteo et al. [14]), we focused on a 5-year window in identifying relevant literature that is also considered to cover current research [26]. In particular, we focused on the period 2016 – 2021. Second, studies had to be published in one of the determined healthcare management journals (see Table 1). Third, in line with Okoli and Schabram [23], and Xiao and Watson [27], articles had to be peer-reviewed to guarantee the quality of included studies. Fourth, as this study aimed to identify potential barriers and facilitators to sustainable implementation of LM, articles had to report on empirical evidence on sustaining LM in an HCO or network of HCOs at team, departmental and/or organisational level. This implies that review studies using empirical sources could be included, but opinion pieces or editorials had to be excluded. We did not make any specific choices related to HCOs (e.g. focus on hospitals, or elderly care and whether organisations were public or private) in order to create a comprehensive overview of barriers and facilitators that impact LM sustainment in healthcare settings in general. Lastly, as most articles on LM in healthcare were found to be published in English [4*], only English articles were considered.

#### Keywords

Multiple literature reviews on LM in healthcare (i.e. [4, 13, 14, 28, 29]) were reviewed to gather relevant keywords for the literature research, resulting in the following search query: (“Lean thinking” OR “Lean management” OR “Lean healthcare” OR “Lean philosophy” OR “Continuous improvement” OR “Lean methods” OR “Lean principles”). Keywords were required to be present anywhere in the article. As for the search string, sustainability or synonyms thereof were not included as keywords, with this approach we hoped to avoid missing potentially relevant studies. As initially, many non-healthcare studies were identified in BMJ Quality & Safety, the search query was adjusted with the addition “AND Healthcare”.

#### Screening and selection

**Fig. 1:**
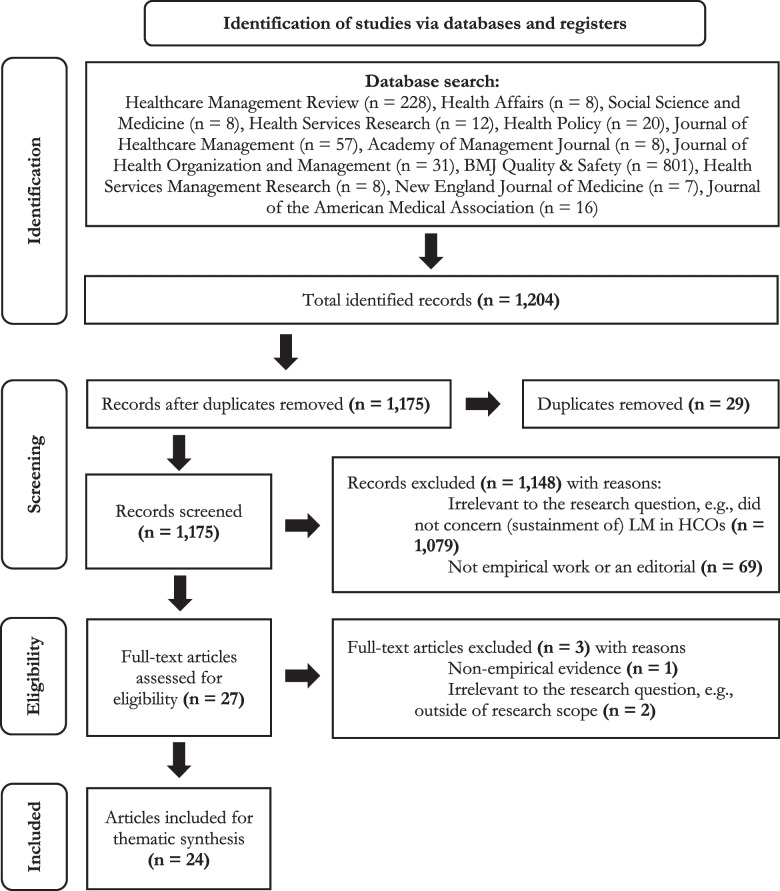
PRISMA-flowchart adapted from Page et al. [21]

We identified a total of 1,204 studies. Figure 1 shows a flowchart of the identified articles. First, 29 duplicate records were removed. Subsequently, the titles and abstracts of the remaining 1,175 articles were manually screened using eligibility criteria to only select relevant articles. A total of 1,148 articles were excluded during the screening stage. In the first round of screening, 1,096 of the 1,148 articles were excluded, as 1,034 were irrelevant to our research question (e.g. no mention of [sustaining] LM in HCOs) and 62 concerned non-empirical works (e.g. editorials or opinions; primarily published in BMJ Quality & Safety). For 59 studies, it was unclear whether articles were suitable for our study, and another researcher was consulted in a second round of screening.

Through consensus, 52 of these articles were excluded, as seven pertained to non-empirically focused works, and 45 articles were irrelevant to our research question. In total, 27 studies remained, for which full-text articles were retrieved. Three articles were excluded after reading full texts. One of the articles concerned non-empirically focused evidence, and two articles were irrelevant to answer our research question and were therefore unfit to answer our research question. Hence, our final sample consisted of 24 articles.

#### Data extraction and data analysis

A data extraction form based on Okoli and Schabram [23] was used to extract descriptive information from each article, piloted before conducting the systematic literature review. Data extracted from articles include the author(s), year of publication, journal, study setting, country, research aim, type of research, main findings, and discussed facilitators or barriers within the article. We then engaged in descriptive analysis, using a coding book (Additional File 1) based a priori on the CI model of Fryer et al. [6] and other identified LM literature. This provided us with an initial guiding framework and conceptual lens, which was expanded upon with inductive coding. Data were synthesised through an iterative process of thematic analysis [30]. Following Fereday and Muir-Cochrane [31], we conducted a hybrid deductive and inductive analysis following their proposed coding stages (see Additional File 2).

The coding process was conducted using the software ATLAS.ti 8 Mac. Guided by Saldaña [32], different researchers coded five articles independently to ascertain coding reliability. The articles were subsequently divided and coded. Weekly meetings were held to discuss progress, new codes, and to resolve coding differences. Through open and axial coding [32], coded excerpts in articles are labelled as facilitators or barriers. Herein, we followed the approach of Azevedo et al. [33*] and defined facilitators and barriers, respectively, as activities, employees or context that encouraged LM sustainment or stalled LM sustainment or hindered LM sustainment. Following this coding process, one of the authors independently analysed and interpreted coded data. Similarly, coded excerpts were grouped to identify common themes. The resulting themes were reviewed and refined to construct themes that were discrete and non-repetitive, but broad enough to not potentially lose coded data.

## Results

This section provides a descriptive summary and characteristics of the included studies. Subsequently, identified barriers and facilitators that contribute to the sustainment of LM in HCOs are presented. An overview of the findings is provided in Table 3.

### Characteristics of included studies

**Fig. 2:**
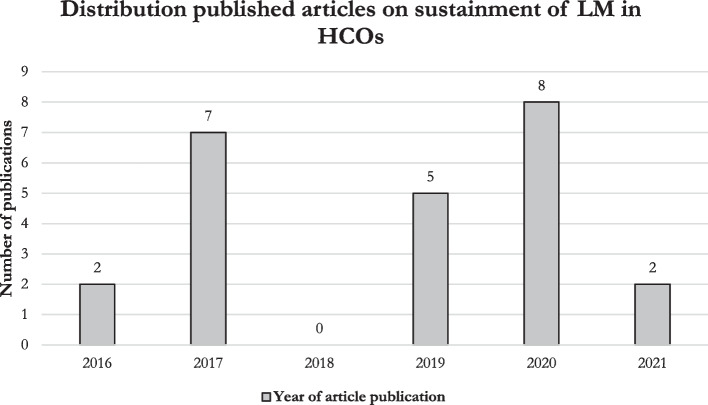
Distribution of publication years within included studies

The largest number of articles were published in the Journal of Health Organization and Management, see Table 2. The distribution of the included articles encompassed various continents. Articles originated from Jordan (*n* = 1, 4%) the United States (*n* = 9, 38%), Brazil (*n* = 2, 8%), Canada (*n* = 1, 4%), The Netherlands (*n* = 4, 17%), New Zealand (*n* = 2, 8%), Sweden (*n* = 2, 8%), and the United Kingdom (*n* = 3, 13%). The highest number of studies were published in 2017 (*n* = 7) and 2020 (*n* = 8), whereas zero publications were observed in 2018 (see Fig. 2). The trend in publications on sustaining LM in HCOs is inconsistent, contrasting the increased trend in LM publications in healthcare as found by Akmal et al. [4, 14]. The most common research methodology observed was qualitative (*n* = 11, 46%), which was expected as Pearce and Pons [26] found that most research on LM is qualitative. Other methodologies include mixed-methods (*n* = 2, 8%), quantitative research (*n* = 5, 21%), and literature reviews (*n* = 6, 25%).

**Table 2:** Article distribution per employed journal

Journal	*n*	*%*
BMJ Quality & Safety	5	21
Healthcare Management Review	4	17
Journal of Health Organization and Management	10	42
Journal of Healthcare Management	3	13
Health Services Management Research	1	4
Health Policy	1	4
Included articles	24	100

The cumulative percentages in the table are rounded off

Further characteristics of the included studies, their main findings and discussed facilitators and barriers have been added in Additional File 3. Four broad themes resulted from the thematic analysis, which encompasses facilitators and barriers that were found to influence the sustainment of LM in HCOs. The identified themes were 1. Mobilising Employees, 2. Guiding Change Efforts, 3. Methods, and 4. Local Context. Table 3 provides an overview of which articles contributed to the themes. In the following sections, the respective themes and identified factors that facilitate or pose a barrier to the sustainment of LM in HCOs are addressed further in detail.

**Table 3:** Overview of the identified (sub)themes described in articles wherein facilitators and barriers influence sustainment of LM in HCOs

Theme	Subtheme (description)	N	Articles addressing this subtheme
1 Mobilising Employees			
1a	Staff empowerment (the extent to which staff is empowered and involved in the decision-making process)	9	[34*, 35*, 36*, 37*, 38*, 39*, 40*, 41*, 42*, 43*]
1b	Staff engagement (the extent to which employees are committed to an organisation)	11	[34*, 35*, 36*, 38*, 39*, 40*, 42*, 44*, 45*, 46*, 47*]
1c	Change agents (early adaptors or innovators in the implementation of an innovation)	6	[33*, 34*, 35*, 47*, 48*, 49*]
2 Guiding Change Efforts			
2a	Leadership (the extent to which leaders are capable to initiate and lead change)	13	[33*, 34*, 35*, 36*, 38*, 39*, 40*, 41*, 42*, 43*, 46*, 47*, 48*]
2b	Management (the extent and way management communicate with and guide employees)	9	[33*, 34*, 35*, 36*, 38*, 39*, 40*, 43*, 50*]
3 Methods			
3a	CI methods (the extent to and way LM practices are present in an organisation)	12	[4*, 34*, 35*, 38*, 41*, 42*, 43*, 44*, 47*, 50*, 51*, 52*]
3b	Scope of CI initiatives (the extent to which LM is applied in an HCO)	10	[4*, 34*, 40*, 43*, 46*, 47*, 50*, 51*, 53*, 54*]
3c	Training and learning (the extent to which learning opportunities are available for staff)	11	[34*, 35*, 36*, 38*, 39*, 43*, 44*, 45*, 47*, 50*, 55*]
4 Local Context			
4a	Organisational resources (the extent to which an organisation has access to [in]tangible resources)	5	[35*, 39*, 45*, 46*, 47*]

#### Theme 1: mobilising employees

Seventeen studies discussed facilitators and barriers within the theme Mobilising Employees that influence sustainment of LM, divided into subthemes staff empowerment (*n* = 9), staff engagement (*n* = 11) and change agents (*n* = 6). An overview of identified facilitators and barriers is provided in Table 4.

**Table 4:** Facilitators and barriers identified within the theme Mobilising Employees that can influence sustainment of LM as reported in studies

Subtheme	Facilitators	Barriers
**1a. Staff empowerment**	• Assigning more responsibilities to workers in the decision-making process [34*, 36*, 37*, 39*, 41*, 42*, 43*]	• Overburdening workers with responsibilities [35*, 38*, 40*]
**1b. Staff engagement**	• Encouragement of (frontline) workers to engage in LM [35*, 36*, 38*, 39*, 42*, 47*]	• Dominance of LM experts in the LM adoption or implementation process [35*]
	• Involvement of frontline staff in the LM design process across disciplines and hierarchical levels [35*, 36*, 39*, 40*, 44*, 46*]	• Strict top down LM approach and omitting staff involvement [35*, 36*, 40*]
	• Participation of physicians in LM [35*]	• Leaders that do not acknowledge the value of employees [34*, 45*]
	• The appeal of LM to care professionals [35*, 39*, 44*]	
**1c. Change agents**	• Appointment of change agents to act as local LM leaders [33*, 34*, 35*, 47*, 48*, 49*]	

1a: In six studies [34*, 36*, 37*, 39*, 41*, 42*], staff empowerment led to a feeling of ownership of process changes, aiding the sustainment of LM through increased staff engagement. The methods used to empower workers varied. Aij and Teunissen [34*] empowered workers through teamwork and meetings, and Schouten et al. [39*] empowered workers by making them partly responsible for planned changes. Three studies [35*, 38*, 40*] report that the overburdening of workers with responsibilities may cause increased work pressure and negatively influence CI project success. Rees and Gauld [38*] recommended using project scheduling to reduce the burden of LM on employees.

1b: Staff engagement influenced the grade to which a culture of CI was present in four studies [35*, 36*, 46*, 47*]. The participation of workers in the LM design process through meetings helped achieve a positive attitude towards LM, resulting in increased staff engagement enabling continuous improvement [36*, 39*, 40*]. Participation of physicians in the study of Harrison et al. [35*] led to increased willingness to adopt process changes. Schouten et al. [39*] and Taylor et al. [44*] found that engagement with LM is more likely when it appeals to professionals’ values. Prolonged staff engagement resulted in a feeling of ‘ownership’ among workers in the study of Hung et al. [36*], stimulating the sustainment of LM. A strict top-down implementation approach to LM led to resistance to change, which reduced staff engagement in three studies [35*, 36*, 40*].

1c: In five studies [33*, 34*, 35*, 47*, 48*], change agents were employed as catalysts to initiate change. Change agents positively influence staff engagement by helping workers to embrace or engage in LM [33*, 47*, 48*]. This finding is complemented by Aij and Teunissen [34*], who report that the workforce and leaders should act as agents to stimulate engagement in LM. Kaltenbrunner et al. [49*] nuance the prior findings and argue that the sheer appointment of change agents does not contribute to the sustainment of LM but is a complementing factor.

#### Theme 2: guiding change efforts

Fourteen studies discussed facilitators and barriers related to the theme Guiding Change Efforts, divided into subthemes leadership (*n* = 13) and management (*n* = 9). An overview of identified facilitators and barriers is added in Table 5.

**Table 5:** Facilitators and barriers identified within the theme Guiding Change Efforts that can influence sustainment of LM as reported in studies

Subtheme	Facilitators	Barriers
**2a. Leadership**	• Encouragement of frontline workers [34*, 36*, 41*, 46*, 47*, 48*]	• Not making resources available for personnel [33*, 38*, 40*]
	• Ability to adopt and blend leadership behaviours and openness to develop leadership competences [34*, 35*, 41*, 42*, 43*]	• Lack of knowledge of LM, lack of commitment from leaders and lack of follow-up on projects [35*, 38*, 39*, 43*]
	• Facilitating open communication between staff [36*, 43*]	• Inability to grasp the need for systemic change [34*]
	• A clear communication plan [34*, 35*]	
**2b** **Management**	• Careful planning of LM initiatives and goal setting [35*, 50*]	• Limited systemic dissemination of LM project results [35*]
	• Knowledge and ability to translate LM practices to staff workers and involving workers in the LM design process [36*, 39*, 40*, 43*]	• Not making resources available for personnel [33*, 38*, 40*]
	• A clear communication plan [34*, 35*]	

2a: In six studies [34*, 36*, 41*, 46*, 47*, 48*], encouragement from leaders facilitated to promote cultural change. Methods of motivation vary, including daily huddles [46*, 47*, 48*], being receptive to feedback [36*], giving workers confidence, and showing them enthusiasm [41*]. In four studies [34*, 35*, 41*, 47*], the ability to blend and adopt leadership styles (transformational, transactional, or participative) improved CI capability of teams. The development of leadership competencies was determined necessary for staff empowerment by Aij and Teunissen [34*] and van Rossum et al. [42*]. Hung et al. [36*] found that open communication between workers was necessary to achieve CI. A clear communication plan was seen as a facilitator to sustain LM in two studies [34*, 35*], see also subtheme 2b. Barriers within leadership include limited follow-up on completed LM projects [35*] or a lack of visible commitment to LM from leaders [39*], which reduces the potentiality to spread LM in HCOs. Three studies [33*, 38*, 40*] found that a lack of resource allocation from either leaders or management to impede sustainment of LM in heavy workload environments.

2b: Régis et al. [50*] found that using performance indicators and the development of process owners contributed to sustainable LM implementation. Knowledge and the ability to translate LM practices to the workforce was observed as a facilitator in three studies [36*, 39*, 40*]. I.e., through translating LM practices to objectives in steering groups consisting of management and the workforce [39*]. A communication plan was seen as a facilitator to sustain LM in two studies [34*, 35*], both from the perspective of leaders and management. Elements of a communication plan include conveying the need for change [34*], making clear expectations of workers [35*] and systemic dissemination of workers’ experiences and LM project results throughout the organisation [34*]. Harrison et al. [35*] reported a barrier resulting from communication and found that limited systemic dissemination of LM project results led to ineffective implementation of new procedures devised by LM teams.

#### Theme 3: methods

Nineteen studies discussed facilitators and barriers within the theme Methods, divided into subthemes CI methods (*n* = 12), scope of CI initiatives (*n* = 8), and training and learning (*n* = 11). An overview of identified facilitators and barriers is shown in Table 6.

**Table 6:** Facilitators and barriers identified within the theme Methods that can influence sustainment of LM as reported in studies

Subtheme	Facilitators	Barriers
**3a. CI methods**	• Integration of LM in strategic planning [35*, 51*]	• Deceptive simplicity of LM tools/methods [52*]
	• Establishing and spreading developed routines through LM in the HCO [34*, 35*, 50*]	• Intensity of mental, physical and emotional effort and increased workload [35*, 38*, 44*]
	• Fostering an organisation-wide improvement and learning culture [4*, 34*, 38*, 41*, 42*, 43*, 47*, 50*]	• Limited follow-up on LM projects post-completion by project team [35*]
**3b. Scope of CI initiatives**	• Holistic structured approach of LM [4*, 34*, 43*, 47*, 50*]	• Lack of patient engagement in the LM process [46*, 51*]
	• Simple departmental or organisational processes [54*]	• Addressing individual issues as opposed to a holistic approach [4*, 40*, 43*, 53*]
**3c. Training and learning**	• Professional developmental opportunities [34*, 35*, 36*, 38*, 39*, 43*, 44*, 47*, 50*, 55*]	• Deeply institutionalised roles and interactions [36*]
	• Hands-on support from external or internal LM experts [35*, 55*]	• Training that is not tailored to a healthcare context [45*, 47*]

3a: The development and fostering of an improvement and learning culture was found to be a cornerstone for sustainable LM implementation in seven studies [4*, 34*, 38*, 41*, 42*, 47*, 50*]. This does not imply that such a culture needs to be present before LM initiatives are launched, but that these should develop and grow over time. Moreover, methods to foster such a culture varied, including belief in improvement [34*], using team improvement suggestions [38*] or work standardisation [38*, 50*]. Integration of stakeholders in strategic LM planning led to increased commitment and financial support for LM initiatives [35*, 51*]. The establishment and spreading of newly developed routines throughout an HCO can lead to sustained organisation-wide improvements [34*, 35*, 50*]. Barriers reported include the deceptive simplicity of LM methods, resulting in decreased learning experiences [52*] and reduced commitment following participation in LM activities because of increased workload and required emotional effort [35*, 38*, 44*].

3b: Four studies [4*, 34*, 47*, 50*] advocate for a holistic, structured approach to LM as opposed to a localised LM approach, where Radcliffe et al. [47*] found that a holistic approach to LM increased engagement of workers with LM. Aij and Teunissen [34*] argue that for LM to succeed, the scope of LM must completely encompass the HCO. These findings are contrasted with two studies, which report that localised LM approaches can provide sustained unit-specific performance improvements [40*, 54*]. Additionally, Poksinska et al. [51*] found that many LM applications do not consider patient needs or preferences for their value definition. This finding is complemented by Po et al. [46*], who identified that insufficient involvement of patients in LM transformation initiatives may constrain the advancement of improving patient outcomes through LM.

3c: Facilitation of training sessions to qualify workers to engage in LM was a facilitator in nine studies [34*, 35*, 36*, 38*, 39*, 44*, 47*, 50*, 55*]. Upon further analysis, this facilitator was reported more often in public healthcare organisations compared to private healthcare organisations. Learning opportunities included training [47*] or multi-day workshops [44*] provided by LM experts. Complementing these findings, two studies [35*, 55*] reported the benefits of employing hands-on support from internal or external LM experts to enable independent engagement in LM activities. Barriers include untailored LM training sessions, which caused a divide between employees and managers, reducing the uptake of LM knowledge [45*, 47*]. Hung et al. [36*] found that the institutionalisation of social and occupational roles of physicians decreased the acceptance of LM, which required shifts in routines to facilitate continuous improvement.

#### Theme 4: local context

Five of the studies discussed facilitators and barriers within the theme Local Context, divided into the subtheme organisational resources (*n* = 5). An overview of identified facilitators and barriers is added in Table 7.

**Table 7:** Facilitators and barriers identified within the theme Local Context that can influence sustainment of LM as reported in studies

Subtheme	Facilitators	Barriers
4a. Organisational resources	• Available tangible and intangible organisational resources to invest in LM [35*, 45*, 47*]	• Lack of available organisational resources to invest in LM or expand LM in the organisation [39*, 46*, 47*]

4b: Available (in)tangible organisational resources were a facilitator for the sustainment of LM in three studies [35*, 45*, 47*]. Harrison et al. [35*] report that prior experience with improvement initiatives aided in the conceptual and operational foundation for LM to succeed. Ability to finance LM initiatives was seen as a facilitator in two studies, as they were able to dedicate full-time staff resources to LM [35*, 47*]. In contrast, the inability to finance LM initiatives was expressed as a barrier in three studies [39*, 46*, 47*]. This barrier was particularly reported in studies concerning public healthcare organisations. In particular, Po et al. [46*] found that hospitals with fewer resources are behind in system-wide LM implementation than institutions with available resources.

## Discussion

This systematic literature review aimed to provide an overview of facilitators and barriers to sustainable LM implementation in healthcare and to construct a conceptual framework that visualises how factors contribute to the sustainment of LM. To summarise, 21 facilitators, and 17 barriers that influence the sustainment of LM were identified within four themes: 1. Mobilising Employees, 2. Guiding Change Efforts, 3. Methods, and 4. Local Context. Subthemes identified that influence sustainment are as follows: 1a. Staff empowerment, 1b. Staff engagement, 1c. Change agents, 2a. Leadership, 2b. Management, 3a. CI methods, 3b. Scope of CI initiatives, 3c. Training and learning, and 4a. Organisational resources. The results indicate the importance of involving and encouraging the workforce to engage and participate in the LM implementation and adoption process.

In addition to identifying (sub)themes, this review highlights the potential relationships between subthemes that influence the sustainment of LM. For example, change agents were found to positively influence staff engagement [33*, 47*, 48*], staff engagement originated through staff empowerment [34*, 36*, 37*, 39*, 41*, 42*], and the scope of the LM approach was seen as an essential facilitator to achieve system-wide sustainment of LM [4*, 34*, 47*, 50*]. Within the subthemes of communication and organisational resources, cases were observed where the absence of a factor resulted in a barrier to sustaining LM. In contrast, the factor acted as a facilitator when present, indicating the twofold nature of facilitators and barriers.

Interestingly, whilst reviews have associated LM with increased operational effectiveness and cost-efficiency [12–14], studies in our sample primarily concerned operational efficiency (i.e. reduced waiting times) whereas financial efficiency is sparsely addressed or as an element of operational efficiency. Moreover, despite growing importance of patient engagement in continuous improvement [56, 57], only two out of 24 studies highlighted lacking patient engagement as a barrier to sustaining LM efforts. However, patients offer unique perspectives on care administration and simultaneously have an inherent interest in safe and effective healthcare [58]. Best et al. [59] stress engaging all stakeholders of healthcare systems, including patients, for achieving widespread healthcare transformation, which may also extend to sustaining lean transformations in healthcare.

**Fig. 3:**
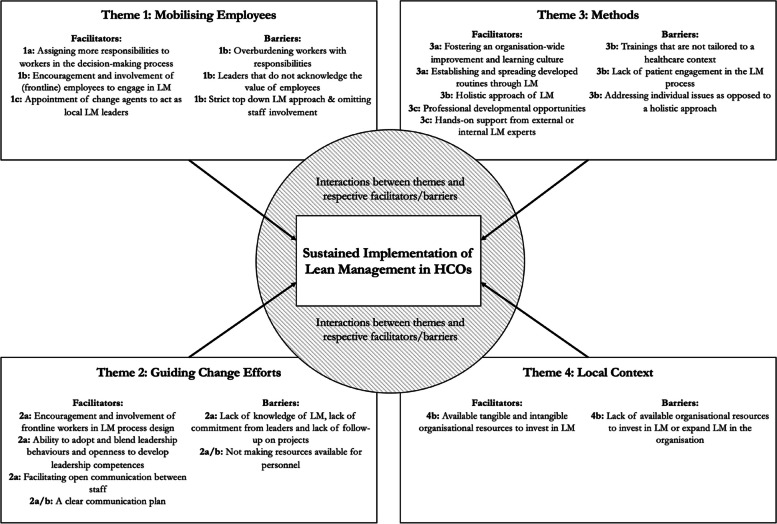
Proposed conceptual framework visualising the key facilitators and barriers, underlying relationships, and their influence on sustainment of LM in HCOs

Based on these findings, we propose a conceptual framework (see Fig. 3) that provides an overview of the identified subthemes, interactions between subthemes, and their connection to sustaining LM in HCOs. Drawing on extant literature, factors that influence the sustainment of LM were predicted to include staff engagement, staff empowerment [7], clear communication, the appointment of change agents, and training [17]. This review supports their findings and contributes additional identified facilitators and barriers in four themes that influence the sustainment of LM in HCOs. Furthermore, the results are in line with classic LM theory [60], supporting the prospect of having respect for people. The findings of this review cover the majority of the three-stage CI model developed by Fryer et al. [6]. However, a discrepancy is observed as their model does not explicitly detail the importance of engaging and involving the workforce in the maturing process, which may be caused by the descriptive nature of their model.

In our study, we did not identify substantial differences between private and public HCOs, albeit the data to identify private and public institutions in the included studies was limited. However, previous research by Radnor et al. [3] identified key contextual differences between private and public institutions (i.e. separation between those who pay for and receive care, and efficient resource reallocation) that may influence factors important in achieving LM sustainment. Hence, we identify this domain as a topic for future studies, as our proposed framework might differ depending on organisational context.

Comparing the findings of this literature review on LM sustainability with published research in other sectors (i.e. manufacturing, furniture, printing), it is evident that noteworthy similarities are observed. I.e. lacking organisational resources, involving employees, effective leadership and management strategies in furniture, manufacturing, and printing industries [61–64]. However, notable discrepancies underscore the unique challenges and considerations specific to adopting and sustaining LM in healthcare. Remarkable distinctive barriers to sustain LM outside healthcare include insufficient government support [63, 64], lack of dedicated supplier(s) [61, 63], and lacking quantitative performance measurement [64, 65]. Differences in reported facilitators and barriers may be explained by the different institutional context of HCOs as service organisations compared to manufacturing organisations [62].

### Implications of this study

The identified barriers, facilitators and conceptual framework can be employed by practitioners who are looking to implement LM in their organisation sustainably. Addressing facilitators and barriers may allow for the sustainment of LM. Moreover, the proposed framework can be used as an addition to the three-stage CI model by Fryer et al. [6]. Using the model of Fryer et al. [6], practitioners responsible for LM implementation can diagnose CI-maturity in their respective HCO(s), whereafter our conceptual framework provides evidence-based insights by showing which elements require strategic attention in fostering a sustainable LM approach. I.e. our framework highlights activities or behaviours that are important for managers and leaders, and it underlines the importance of a holistic approach when adopting LM. Ultimately, the themes provide practitioners with clear elements in their work environment that require attention.

This review provides a theoretical contribution to the literature by providing a comprehensive overview of facilitators and barriers that influence the sustainment of LM in HCOs, which did not exist prior to this study [3, 13, 14]. Additionally, our framework suggests potential relationships between subthemes and achieving sustainment of LM. Moreover, our study highlights omissions in our current understanding of LM in healthcare contexts. In our findings, we did not identify the role of politics in shaping the healthcare context. However, previous studies did emphasise the highly political environment healthcare subsides (i.e. the [financial] efficiency agenda) in Radnor et al. [3], and we would assume this shaping influences organisational change such as LM. Nevertheless, our results did not show any attention to the role politics has for local LM sustainability.

From an academic perspective, the framework offers building blocks that could be used to structure future quantitative studies. For example, survey research could focus on the strength of the relationship between the identified themes and LM sustainability. Alternatively, studies could aim to identify to which degree the various underlying elements (e.g. a clear communication plan) are required to obtain a sustained LM implementation.

### Limitations & future research directions

Notwithstanding the findings, limitations to this review need to be considered. Whilst this review has identified facilitators and barriers in various contexts, it does not provide an exhaustive list. It is possible that factors contributing to the sustainment of LM exist that were not observed. Due to a lack of data on HCOs in Africa and Asia, our findings may not be generalisable to HCOs in those contexts. Though variances in coding between researchers were accounted for, reliability during the coding process might have been increased with more formal intercoder agreement analysis. In addition, employing journals as a proxy for quality has limitations [66] which could have been addressed by conducting a methodological quality assessment [67]. Though excluding articles based on methodology is generally not recommended [23, 67], quality assessment could have provided insight into the quality of studies and enabled a sensitivity analysis [67]. Moreover, in our study we broadly distinguished between staff with leader or managerial responsibilities and frontline employees. However, we do recognise that healthcare staff is highly heterogeneous, and that responses to organisational change may differ. Although our conceptual framework provides generalised facilitators and barriers for sustaining LM, it is not unlikely that specific barriers are especially important for specific professional groups.

Future research directions include expanding the proposed framework to additional aspects of healthcare systems (e.g., nursing homes), and identify if the framework should be adapted to cater to public and private institutions. Furthermore, future studies could focus on identifying facilitators or barriers to sustaining LM in HCOs throughout Asia and Africa. In addition, the future studies could consider the importance of political factors on the implementation of LM in healthcare. Moreover, during our review we identified two sources that reported on patient engagement. Given the centrality of patients in care processes, it seems that patient engagement in relation to LM is an underinvestigated avenue of research. Lastly, we make a limited distinction between healthcare staff and mainly focus on frontline employees, leaders, and managers. Future studies might investigate if LM sustainability is influenced differently when accounting for the various professions (e.g. nurse, physician, support staff, etc.).

## Conclusion

This qualitative systematic literature review sought to identify and aggregate known barriers and facilitators that contribute to the sustainment of LM in healthcare. Following thematic analysis, four overarching themes were identified: Mobilising Employees, Guiding Change Efforts, Methods, and Local Context, wherein 21 facilitators and 17 barriers to sustaining LM were identified. Overall, studies emphasise the importance of actively involving and engaging the workforce to embed LM into organisational culture such that improvement practices are sustained. Reflecting on the origins of LM, healthcare organisations can find inspiration in the virtue of respecting people in their journey to sustain and cultivate an improvement culture.

### Supplementary Information

**Additional file 1: Table A1.** Codebook employed in the thematic coding process.

**Additional file 2: Figure A1.** Illustrative depiction of the stages that were passed throughout the thematic data coding process, adapted from the work of Fereday and Muir-Cochrane [31].

**Additional file 3: Table B1**. Summary of the characteristics and relevant findings of included articles for the literature review. A cross (X) indicates that a facilitator or barrier was not mentioned in an article.

## Data Availability

Supplementary data are included in the additional files.

## Abbreviations

LM: Lean Management
HCOs: Healthcare organisations
CI: Continuous improvement
PRISMA: Preferred Reporting Items for Systematic Reviews and Meta-Analysis
