# Supplementary material for: What are barriers and facilitators in sustaining lean management in healthcare? A qualitative literature review
Source: BMC Health Serv Res. 2023 Sep 6;23:958. doi: 10.1186/s12913-023-09978-4 (PMC10483794; doi:10.1186/s12913-023-09978-4)
Supplement: Supplementary file 1 — Additional file 1: Table A1. Codebook employed in the thematic coding process. [file 12913_2023_9978_MOESM1_ESM.docx]

# Additional File 1: Codebook employed throughout the thematic analysis

| **Table A1:** Codebook employed in the thematic coding process. | | |
| --- | --- | --- |
| **• Code** | **• Definition** | **• Example** |
| Change agent (n=173) | Individuals that participated in the Lean in healthcare application process with varying knowledge of Lean, their different application/implementation approaches and associated effects | *‘Development of clinical “champions” and mid-level managers is a key element for successful, sustained Lean intervention.’* Naik et al. [17 p. 9c5] |
| Communication (n=35) | Types of verbal and non-verbal communication that affect or may affect the process of applying Lean to healthcare settings | *‘Lack of adequate communication sometimes blocked effective implementation of steps recommended by [Rapid Improvement Event] teams.’* Harrison et al. [36 p. 139] |
| Continuous  improvement  (n=87) | A culture wherein all members of an organisation work together on an ongoing basis improving processes and reducing errors to improve overall performances | *‘Integration of CI into the organisation is an indicator of CI. Extensive and widely distributed learning behaviour is stage in which CI is fully integrated.’* Fryer et al. [6 p. 484] |
| Culture (n=108) | The ideas, customs, and social behaviour of a particular people or society | *‘Flexibility and individual initiative cannot be ascribed to a healthcare setting's hierarchy without the visible and sustained support of the senior executive.’* Al-Balushi et al. [28 p. 142] |
| Finance (n=37) | Matters related to finance were mentioned as a result of Lean adoption | *‘Implementing Lean programs required a considerable investment of employee time. Chronic staffing shortages due to turnover were reported and made it difficult for staff to find the time for a robust Lean program.’* Azevedo et al. [33 p. 9] |
| Leadership (n=194) | The action of leading a group of people OR an organisation | *‘This comment illustrates the value of leaders who interact with frontline staff and gain an appreciation for the day-to- day work that is involved in implementing Lean.’* Azevedo et al., 2020 [33 p. 5] |
| Lean combination (n=38) | Combination of Lean with other theories, like Six Sigma or Supply Chain Management | *‘Shah and Ward (2003) were the first to recognize the four lean bundles model consisting of total quality management (TQM), human resource management (HRM), the just-in-time (JIT) system and total productive maintenance (TPM), which have proved successful in manufacturing.’* Abdallah and Alkhaldi [44 p. 488] |
| Lean definition (n=68) | A definition explicitly mentioned in studies regarding what Lean entails | *‘… we define Lean as an overall management/operating system that uses a continuous improvement culture that empowers frontline workers to solve problems and eliminate waste by standardizing work to improve the value of care delivered to patients.’* Rundall et al. [41 p. 11] |
| Lean scope (n=100) | The extent to which lean tool types and the frequency of those tools are applied | *‘We further quantify the scope by analyzing Lean tool types and frequency, and the wastes addressed’* Hallam and Contreras [12 p. 687] |
| Lean tool (n=142) | Lean methods used in a healthcare organisation | *‘Whereas all of them mentioned the tools being used to facilitate the lean implementation process’* Akmal et al. [4 p. 624] |
| Lean objective (n=65) | Aim of Lean implementation or adoption | *‘Lean in health care serves to eliminate waste in processes and create value for patients across the continuum of care’* Azevedo et al. [33 p. 2] |
| Staff empowerment (n=69) | Allows employees to make more decisions for themselves | *‘Both studies stressed leadership commitment to the change and employee empowerment as key instruments in the success of [Lean thinking]’* Akmal et al. [4 p. 621] |
| Staff engagement (n=131) | Staff engagement is the extent to which employees feel passionate about their jobs, are committed to the organisation, and put discretionary effort into their work | *‘As showed by previous studies of the same hospital, efforts to integrate operational and sociotechnical improvement activities with the kaizen system may lead to a better understanding of the relationship between work and health and a higher engagement in health promotion, as well as more engagement in using kaizen for improvement work in general.’* Mazzocato et al. [29 p. 7] |
| Training  and learning  (n=97) | Any event that encompasses training or learning individuals about Lean tools or methodologies throughout an organisation | *‘There was lots of training, lots of workshops, lots of sort of self-assessment workshops, lots of facilitated self-assessments so that each business unit was undertaking its own assessment and then identifying the way forward’* Fryer et al. [6 p. 488] |

*Note.* Abbreviation list*:* CI, *Continuous Improvement*; HRM*, Human Resource Management;* JIT*, Just-in-Time;* TPM*, System and Total Productive Maintenance;* TQM*, Total Quality Management.*
