# Supplementary material for: What are barriers and facilitators in sustaining lean management in healthcare? A qualitative literature review
Source: BMC Health Serv Res. 2023 Sep 6;23:958. doi: 10.1186/s12913-023-09978-4 (PMC10483794; doi:10.1186/s12913-023-09978-4)
Supplement: Supplementary file 2 — Additional file 2: Figure A1. Illustrative depiction of the stages that were passed throughout the thematic data coding process, adapted from the work of Fereday and Muir-Cochrane [31]. [file 12913_2023_9978_MOESM2_ESM.docx]

# Additional File 2: Codebook employed throughout the thematic analysis

Stage 1: Development of a coding book

Stage 2: Checking reliability of deductive codes

Stage 3: Data summarisation and discerning initial themes

Stage 4: Application of the coding book and inductive coding

Stage 5: Connecting codes and identifying themes

Stage 6: Substantiation and legitimation of coded themes

**Figure A1:** Illustrative depiction of the stages that were passed throughout the thematic data coding process, adapted from the work of Fereday and Muir-Cochrane [31].
