# Supplementary material for: What are barriers and facilitators in sustaining lean management in healthcare? A qualitative literature review
Source: BMC Health Serv Res. 2023 Sep 6;23:958. doi: 10.1186/s12913-023-09978-4 (PMC10483794; doi:10.1186/s12913-023-09978-4)
Supplement: Supplementary file 3 — Additional file 3: Table B1. Summary of the characteristics and relevant findings of included articles for the literature review. A cross (X) indicates that a facilitator or barrier was not mentioned in an article. [file 12913_2023_9978_MOESM3_ESM.docx]

| Additional File 3: Characteristics of included studies |
| --- |

| **Table B1:** Summary of the characteristics and relevant findings of included articles for the literature review. A cross (X) indicates that a facilitator or barrier was not mentioned in an article. | | | | | |
| --- | --- | --- | --- | --- | --- |
| **• Author(s) (year)**  **• Journal** | **• Country, study setting**  **• Research aim** | **Research method** | **Main findings** | **Facilitators in study** | **Barriers in study** |
| Abdallah and Alkhaldi [44]  • Journal of Health Organization and Management | • Jordan, scoping review  • Identify research gaps and provide recommendations for future research on LM in healthcare | Literature review | Most studies demonstrated application of selective LM practices in healthcare as opposed to LM bundles, which comprehensively reviews LM and all of its aspects in a multidimensional approach. Adopting LM bundles (i.e. TQM, HRM, JIT, and TPM) may aid HCOs to maximise their performance. | 1a, 1b, 2a, 2b, 3a, 3b, 3c | 2a, 3b |
| • Aij and Teunissen [35]  • Journal of Health Organization and Management | • NL, systematic literature review  • Identify ideal leadership attributes to achieve LM in HCOs | Literature review | Attributes of Lean leadership values were identified that can aid to achieve desired outcomes concerning workers and processes in an organisation. | 1a, 1c, 2a, 2b, 3a, 3b, 3c | 1b, 2a |
| • Akmal et al. [4]  • Health Policy | • NZ, systematic literature review  **•** Identify core LM characteristics and find the relationship between LM and HSCM | Literature review | This study has found that LM in healthcare is still in its infancy. Furthermore, a lack of system wide LM implementation in healthcare, which contradicts the core LM principles was highlighted in this study. | 3a, 3b | 3b |
| • Azevedo et al. [33]  • Health Care Management Review | **•** US, multi-case study in ten Veterans Health Administration medical centres **•** Evaluate barriers and facilitators to LM implementation | Qualitative | A cultural transformation was achieved during LM implementation, which was facilitated through careful planning, scoping, pacing and implementing accountability measures. This study has demonstrated the importance of training and coaching to cement LM in daily routine. | 1c | 2a, 2b |
| • Card [53]  • BMJ Quality & Safety | **•** US, narrative review  **•** Discuss limitations of the ‘5 Whys’ method in root cause analysis in HCOs | Literature review | Results indicate that the use of ‘5-Whys’ is unfit for RCA in patient safety analysis in the healthcare context. The perceived simplicity of LM tools may provide a barrier for understanding of actual inner workings and for change to occur. | X | 3a |
| • Esper et al. [49]  • Journal of Healthcare Management | **•** US, multi-case study in Emory Healthcare (academic medical centre with 11 hospitals and more than 250 clinical sites) **•** Rapid implementation of Outpatient Telehealth in Emory’s physician group practice using Lean | Qualitative | A rapid system-wide deployment of an artifact was achieved by employing a standardised approach to LM. Various elements of LM were crucial in achieving this feat including leadership, daily huddles and standardised work. | 1c, 2a | X |
| • Harrison et al. [36]  • Health Care Management Review | **•** US, multi-case study in five hospital systems **•** To further researchers’ awareness of internal organisational conditions and processes that can affect implementation of LM and other improvement initiatives | Qualitative | By analysis of 5 HCOs that deployed LM in their operations, multiple contextual factors were identified that facilitated or hindered LM project success throughout 8 constructed domains. | 1b, 1c, 2a, 2b, 3a, 3c, ~~4~~b | 1a, 1b, 2a, 2b, 3a |
| • Hung et al. [37]  • Health Care Management Review | **•** US, case study in a large, not-for-profit, ambulatory care delivery system **•** Identify contextual factors most critical to implementing and scaling LM redesigns across primary care clinics | Qualitative | Contextual factors were identified that influence the level of acceptance among frontline primary care physicians and staff. Contextual factors were categorised as (1) the implementation process, (2) inner setting and (3) individual and team characteristics. | 1a, 1b, 2a, 2b, 3c | 1b, 3c |
| • Kaltenbrunner et al. [50]  • Journal of Health Organization and Management | **•** SWE, mixed methods study (survey study n=298 in 45 primary care units, multi-case study observation in 4 primary care units) **•** Describe LM maturity in primary care and to determine the extent to which Lean maturity is associated with quality of care | Mixed-methods | Found that the maturity of LM in primary care units varied concerning adoption of LM principles and LM adoption between primary care units. Maturity of LM adoption was positively associated with quality of care. | 1c | X |
| • Kellogg et al. [54]  • BMJ Quality & Safety | **•** US, case-study in large, tertiary care academic medical centre (750 beds) **•** Aims to build on prior work by examining the types of solutions proposed in response to RCAs | Qualitative | Observed that certain types of events reoccur despite recommendations from RCA teams. Results have concluded that devised solutions for identified problems in healthcare (in particular patient safety) via RCA may not be effective compared to other industries. | X | 3b |
| • Kreindler [55]  • BMJ Quality & Safety | **•** CA, case study in a regional health system  **•** Identify common design flaws that limit the impact of (patient) flow Lean improvement initiatives in healthcare | Qualitative | Concluded that initiatives to improve flow of patients focus on too small segments to properly address shortcomings. Instead, they suggest a system-level strategy is required to improve flow in healthcare, which can be achieved by rigorously evaluating proposed flow initiatives in terms of population, capacity and process. | 3b | X |
| • McNicholas et al. [56]  • BMJ Quality & Safety | • UK, case study in the National Institute for Health Research Collaboration for Leadership in Applied Health Research and Care  • Assess fidelity of PDSA cycles, determine changes in PDSA fidelity over time and explore strategies used to deploy PDSA | Quantitative | Demonstrated that fidelity of PDSA can improve over time through the deployment of quality improvement support strategies. They found that quality improvement technologies should be considered sociocultural interventions, which require technical skill. The findings suggest that the use of PDSA can be increased through the support strategies, though it is a gradual process which requires negotiation. | 3c | X |
| • Menachemi et al.  [38]  • Journal of Healthcare Management | • US, pooled cross-sectional study in the Indiana University Healthcare system (16 hospitals) **•** Analyse the experience implementing LM Rapid Improvement Events across a complex mix of settings in one system. | Quantitative | Financial returns following a system wide implementation of LM were observed across various healthcare settings. Five years after LM implementation, cost savings were the most reported benefit. Additional realised benefits include reduction in clinical defects, optimised workflows and time savings. | 1a | X |
| • Po et al. [47]  • Journal of Healthcare Management | **•** US, survey study (n=1,222 acute care general medical and surgical hospitals) **•** Examining important independent performance measures between LM adoption by 2014 and performance measures in 2015 | Quantitative | Results provide a benchmark for adoption of LM in both public and private hospitals. In particular, the extent of LM adoption is observed to be lower for public hospitals compared to non-profit or for-profit hospitals. The most common LM practices employed were daily huddles, PDSA cycles, visual management and standardised work. | 1b, 2a | 3b, 4a |
| • Poksinska et al. [52]  • BMJ Quality & Safety | **•** SWE, quantitative study (survey via National Patient Survey, n=2400-2800) and two case studies (two primary care centres) **•** Investigate how primary-care centres working with Lean define and improve value from the patient’s perspective, and how the application of Lean healthcare influences patient satisfaction | Mixed-methods | Results indicated that, despite suggestions from literature, patient satisfaction did not increase in primary care centres that work with LM. This may be an indication for the limited scope in which LM is operationalised in HCOs, as tools insufficiently enhance customer participation in the improvement process. | 3a | 3b |
| • Radcliffe et al. [48]  • Journal of Health Organization and Management | **•** UK, ethnographic study in an NHS teaching hospital in medical imaging departments **•** Identify barriers and enablers to Lean implementation as part of an imaging quality improvement programme from a socio-cultural perspective. | Qualitative | Barriers and enablers to LM implementation in healthcare were identified in a complex HCO setting. Three overarching themes were determined: (1) change agents and interprofessional relationships, (2) moving toward a cultural transition and (3) staff engagement and facilitator credibility. | 1b, 1c, 2a, 3a, 3b, 3c, 4a | 3c, 4a |
| • Rees and Gauld [39]  • Journal of Health Organization and Management | • NZ, general review **•** Discuss the effects of the introduction of Lean into HCOs concerning work intensification | Literature review | Results observed that much of the LM literature explores LM implementations with little attention paid to potential effects on cultural and situational factors. These factors, if unaddressed, may lead to work intensification for vulnerable workers. | 1b, 3a, 3c | 1a, 2a, 2b, 3a |
| • Régis et al. [51]  • Journal of Health Organization and Management | **•** BR, three case studies conducted in three hospitals  **•** Develop a practice-driven methodology for implementing Lean in hospital operations | Qualitative | Researched the implementation process and proposed a methodology for implementation of LM in a two-step process: preparation and application. The methodology is based on empirical findings that forms a roadmap for HCOs to employ during the LM implementation process. | 2b, 3a, 3b, 3c | X |
| • Rundall et al. [41]  • Health Care Management Review | **•** US, survey study, filled out by n=1,152 hospitals  **•** Examine the relationships between LM and hospital financial performance, patient outcomes, and patient satisfaction | Quantitative | Demonstrated that the adoption of LM is not significantly linked with 9 out of 10 commonly used measures of hospital performance indicating that adoption of LM may not be associated with improved organisational performance. Localised LM approaches were observed to provide benefits, where they found improvements on a hospital wide measure of performance requires sustained LM. | 1b, 2b | 1a, 1b, 2a, 2b, 3b |
| • Santos et al. [46]  • Journal of Health Organization and Management | **•** BR, systematic literature review **•** Understand the state of the art of LM in healthcare by investigating and comparing conceptual and analytical articles | Literature review | Results indicate that the Visual Stream Map is the most commonly discussed LM tool, and the first tool commonly applied in HCOs. Additional results include that the working environment and management are crucial for LM implementation. Both categories contain many facilitators and barriers to LM. Emergency departments are the most discussed department in articles and performance indicators had a positive effect on LM outcomes. | 4a | 1b, 3c |
| • Schouten et al. [40]  • Journal of Health Organization and Management | **•** NL, case study conducted in Zaans Medical Centre  **•** Investigate key mechanisms enhancing healthcare professionals’ participation and collaboration in implementing Lean-led hospital design innovative approach | Qualitative | Three key mechanisms were identified that caused healthcare professionals to participate and collaborate in LM process redesign of hospitals. The mechanisms were (1) freedom to translate a concept, (2) a set of guiding LM principles and (3) co-creation which was achieved through multi-disciplinary workshops. | 1a, 1b, 2b, 3c | 2a, 4a |
| • Taylor et al. [45]  • Journal of Health Organization and Management | **•** UK, case study performed in a Foundation Trust Hospital  **•** The purpose of this study was to explore individuals’ perceptions and emotions and find barriers/facilitators may influence LM implementation | Qualitative | The results demonstrate that not only adherence to LM values and prescribed steps in LM activities initiate and sustain the improvement process, but also the individuals’ perceptions and emotions are fundamental to sustain LM. A humanistic framework was proposed to provide guidelines to follow during LM workshops to acknowledge the human factor in participation of LM. | 1b, 3c | 3a |
| • van Elp et al. [42]  • Health Services Management Research | **•** NL, case study performed in an anonymised hospital  **•** Identify suitable leader behaviours in healthcare teams to achieve CI in HCOs | Qualitative | This study has identified that a hybrid leadership style is required, combining transactional and transformational principles, to improve continuous improvement capabilities of teams. | 1a, 2a, 3a | X |
| • van Rossum et al. [43]  • Journal of Health Organization and Management | **•** NL, cross sectional study performed in a Dutch university medical centre  **•** Increase scientific knowledge regarding factors that diminish the implementation gap of LM in HCOs from a Change Management perspective | Quantitative | Transformational leadership at the senior management level, team leadership and workforce flexibility were identified as crucial factors for transformation of a department to lean healthcare. In particular, the leadership style stimulated top-down commitment from management and enabled professional autonomy. An additional finding is that the workforce flexibility determines to what extent organisational elements can be adjusted to allow for implementation of LM across departments. | 1a, 1b, 2a, 3a | X |
| *Note.* Abbreviation list*:* BR*, Brazil;* CA*, Canada;* CI*, Continuous Improvement;* HCOs*, Healthcare Organisations;* HRM*, Human Resource Management;* JIT*, Just-in-Time;* LM*, Lean Management;* NL*, The Netherlands;* NZ*, New Zealand;* PDSA*, Plan-Do-Study-Act;* RCA*, Root Cause Analysis;* SWE*, Sweden;* UK*,* TPM*, System and Total Productive Maintenance;* TQM*, Total Quality Management; UK, United Kingdom;* US*, United States.* Subthemes: 1a. *Staff empowerment,* 1b. *Staff engagement*, 1c. *Change agents*, 2a. *Leadership*, 2b. *Management*, 3a. *CI methods*, 3b. *Scope of CI initiatives,* 3c. *Training and learning*, 4a. *Organisational resources*. | | | | | |
